# Effect of Phenolic Resin Oligomer Motion Ability on Energy Dissipation of Poly (Butyl Methacrylate)/Phenolic Resins Composites

**DOI:** 10.3390/polym12020490

**Published:** 2020-02-23

**Authors:** Xing Huang, Songbo Chen, Songhan Wan, Ben Niu, Xianru He, Rui Zhang

**Affiliations:** 1 School of Materials Science and Engineering, Southwest Petroleum University, Chengdu 610500, China; huangxing0226@foxmail.com (X.H.); chen756424838@foxmail.com (S.C.); songhanwan@foxmail.com (S.W.); ben.niu.1@foxmail.com (B.N.); 2 Institute für Physik, Universität Rostock, Albert-Einstein-Str. 23-24, 18051 Rostock, Germany; rui.zhang@uni-rostock.de

**Keywords:** polymer-matrix composites, molecular weight, dynamic mechanical properties, molecular motion, hydrogen bonding

## Abstract

Poly (butyl methacrylate) (PBMA) was blended with a series of phenolic resins (PR) to study the effect of PR molecular weight on dynamic mechanical properties of PBMA/PR composites. Differential scanning calorimetry (DSC) and dynamic mechanical analysis (DMA) found a similar variation of glass transition temperature (T_g_). The maximum loss peak (tanδ_max_) improved in all PBMA/PR blends compared with the pure PBMA. However, tanδ_max_ reduced as the molecular weight increased. This is because PR with higher molecular weight is more rigid in the glass transition zone of blends. The hydrogen bonding between PBMA and PR was characterized by Fourier transform infrared spectroscopy (FTIR). Lower molecular weight PR formed more hydrogen bonds with the matrix and it had weaker temperature dependence. Combined with the results from DMA, we studied how molecular weight affected hydrogen bonding and thus further affected tanδ_max_.

## 1. Introduction

In order to reduce the harm caused by vibration in people’s daily life and industrial production, the development of damping materials is becoming more and more important [1]. Polymer damping materials, as a kind of passive damping material, have attracted wide attention because it is simpler to implement and more cost-effective than semi-active and active techniques [2]. When vibration and noise are generated, it can convert mechanical energy into heat energy through internal friction between molecule around its T_g_. However, in practical application, polymer damping materials are usually required to play a high damping capacity in a specific temperature range. In order to achieve this goal, some methods like blends, interpenetrating networks, and copolymerization were adopted [3,4,5,6,7,8]. Among them, blending is an effective and simple method to regulate the damping properties of materials.

Some scholars have tried to combine polymers with organic or inorganic fillers in order to obtain high performance polymer damping materials. Yin et al. blended petroleum resin with chlorinated butyl rubber (CIIR), thus improving its damping temperature range. The contact angle experiments revealed that van der Waals interactions play an important role in improving the damping properties [9]. Wu et al. [10] found that OMMT form a filler network in CIIR/MMT composites which can suppresses the maximum of loss peak and have little influence on the glass transition temperature. Jiang [11] got different results by blending GO with CIIR. The existence of GO reduced the damping temperature and he attributed this phenomenon to the two-dimensional layered structure of GO. It is obvious that the interaction between polymers and fillers have a great influence on the damping properties. In fact, a strong adsorption effect between fillers and matrix increases T_g_. On the contrary, weak interaction may reduce T_g_ [12].

Compared to T_g_, tanδ_max_ is the focus of this paper. Tanδ_max_ is influenced by molecular motions on different length scales and it is difficult to improve on the high temperature side. Wu [13] found that hydrogen bonding between polymer and small molecule substance can effectively improve tanδ_max_. This provides a new idea for the preparation of high performance damping materials. In recent years, some reports about the effect of hydrogen bonding on the damping properties of polymer/small molecule hybrids have been reported [14,15]. Some scholars [16,17] have prepared composites with high damping properties by blending polymer with inorganic materials grafted with hindered phenol. However, there are also some examples showing hydrogen bonds do not increase tanδ_max_ [18,19]. This phenomenon aroused our concern and promoted our work.

Hydrogen bonding is a kind of reversible dynamic physical bonding. The energy dissipation from it should be affected by the motion ability of filler molecules. T_g_ of the fillers can reflect its motion ability and it increases with the increase of molecular weight for polymer oligomers. Therefore, it is theoretically possible to adjust the motion ability of fillers by changing its molecular weight.

Poly (butyl methacrylate) (PBMA) is a homologue of acrylate polymers, which is widely used as pressure-sensitive adhesives, paints, coatings, seals, and damping elements [20]. However, it has a T_g_ close to room temperature, about 20 °C. This characteristic makes it have better application prospect in higher temperature range. PR is formed by condensation of some simple low molecular weight compounds and is widely used in molding powders, laminating resins, adhesives, binders, surface coatings due to its lower manufacturing cost, dimensional stability, chemical resistance, and thermal stability [21,22]. PR can form hydrogen bonds with polymer matrix because of its high density of hydroxyl groups. In recent years, some researchers have applied phenolic resin for the preparation of polymer-based damping materials [23]. It was found that the chemical structure of phenolic resin have great influence on the damping properties. However, few people have studied the effect of molecular weight.

In this paper, five kinds of PR with different molecular weights were blended with PBMA by solvent blending. The dynamic mechanical behavior and segment dynamics were measured by DSC and DMA. An interesting variation about tanδ_max_ was discovered. FTIR was used to analyze the interaction between PR and PBMA. Finally, we put forward explanation to interpret the phenomenon observed in our case.

## 2. Experimental

### 2.1. Materials

**Figure 1:**
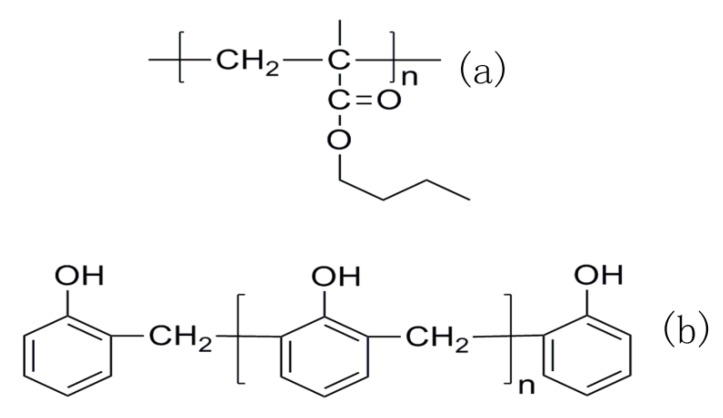
Chemical structures of (**a**) poly (butyl methacrylate); (**b**) phenolic resin.

PBMA was synthesized by emulsion polymerization in our laboratory. A series of linear phenolic resins (558, 8218, PR-2, m01, 8219) were provided by Chengdu Keyi Polymer Technology Co., Ltd. (Chengdu, China). The chemical structures of PBMA and PR are shown in Figure 1a,b, respectively. For the sake of discussion, PR is recorded as A01, A02, A03, A04, and A05 according to their molecular weights from small to large. The blends are recorded as B01, B02, B03, B04, and B05 according to the corresponding phenolic resin. The details are listed in Table 1. The PBMA/PR composites were prepared by solvent blending at a mass ratio of 50/50. PBMA was also blended with A04 at a blending ratio of 90/10 90/20 to study the effect of PR contents on damping performance. Sodium dodecyl benzene sulfonate, potassium persulfate, anhydrous ethanol, neutral alumina, ethyl acetate, butyl methacrylate were purchased from Chengdu Kelong Chemical Reagent (Chengdu, China).

### 2.2. Preparation of PBMA via Emulsion Polymerization

BMA was passed through neutral alumina columns before use to remove the polymerization inhibitor. In the first step, 160 mL deionized water, 100 mL BMA monomer, 4 g emulsifier were added into a 500-mL three-necked flask. Nitrogen was stirred in at room temperature for 20 min and then heated to 72 °C. At the same time, 0.3 g initiator was dissolved in 40 mL deionized water and dripped into the emulsion through a constant pressure drip funnel. When the liquid surfance appears light blue, the reaction was continued for 4 h. The product was precipitated with anhydrous ethanol. Then filtered with a vacuum pump until the liquid in the lower layer is clarified. The product is then placed on a evaporation dish and dried in a vacuum oven.

### 2.3. Preparation of PBMA/PR Composites via Solvent Blending

Ethyl acetate was selected as the co-solvent for PBMA and PR. First, PBMA (3 g) and PR (3 g) were dispersed in 50 mL of ethyl acetate at room temperature for 8 h to form an even solution. Then 250 mL of deionized water was added to precipitate the sediment. The sediment was collected by watch glass and then dried in a vacuum oven at 80 °C for 24 h.

### 2.4. Characterization

FTIR was carried out on Nicolet 6700 (Thermo Fisher Scientifc, Waltham, MA, USA). Total of 0.2 g blends was dissolved in 2 mL tetrahydrofuran (THF) and smeared on the CaF_2_ windows. After the solvent is completely volatilized, the sample was fixed and tested. For the temperature scans, the samples were placed in a heating cell connected to a temperature controller. The temperature range was from 30 °C to 170 °C. The carbonyl stretching region was fitted by two Gaussian bands, corresponding to free and hydrogen bonded carbonyl band near 1728 cm^−1^ and 1704 cm^−1^, respectively.

DSC test was performed on DSC-822^e^ (Mettle Toledo, Zurich, Switzerland). The sample was first heated from −20 °C to 120 °C at a rate of 15 °C/min and then cooled toward −20 °C at the same rate. Finally, a heating process at a heating rate of 3 °C/min to 120 °C was performed to measure the T_g_. For PR, we are going to approximate its softening point as T_g_ because its molecular weight is so small that there is no rubber platform.

DMA was carried out on Q800 (TA Instruments, New Castle, DE, USA) by using a dual cantilever clamp and a testing method of temperature step-frequency sweep under a heating rate of 5 °C/min within a temperature range of −20 °C to 200 °C and a frequency of 1 Hz.

## 3. Results and Discussion

### 3.1. Structure Characterization of Blends and PBMA

**Figure 2:**
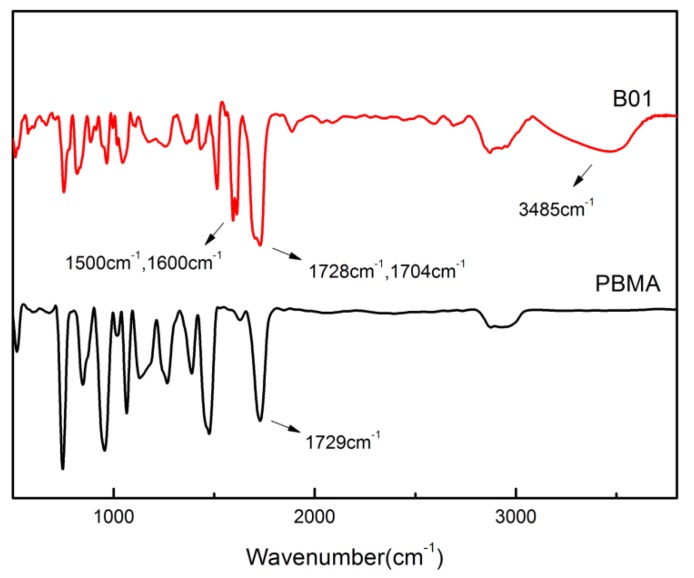
Infrared spectra of poly (butyl methacrylate) (PBMA) and B01.

The FTIR spectra were used to characterize the structures of PBMA and B01. As shown in Figure 2, the characteristic absorption peak at 1729 cm^−1^ can be attributed to the stretching vibrations of carbonyl. B01 shows the characteristic absorption peaks at 1500 cm^−1^ and 1600 cm^−1^ which can assign to the stretching vibrations of conjugate double bonds in aromatic ring framework. The characteristic absorption peak of hydroxyl appeared at 3485 cm^−1^ and the peak of carbonyl splits into two peaks located at 1704 cm^−1^ and 1728 cm^−1^ respectively for B01. These phenomena indicate the formation of hydrogen bonding between PR and PBMA.

### 3.2. Glass Transition of PBMA/PR Composites

**Figure 3:**
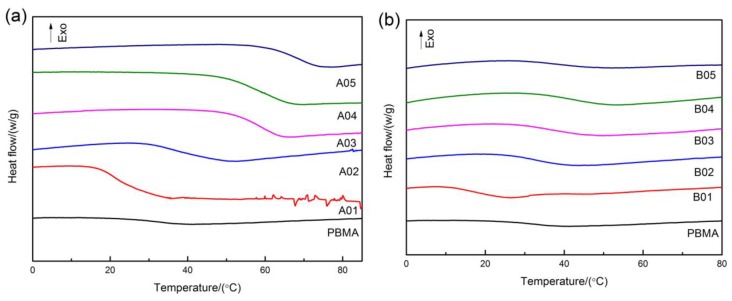
The differential scanning calorimetry (DSC) thermograms of PBMA, (**a**) PR with different molecular weights and (**b**) PBMA/PR blends.

**Figure 4:**
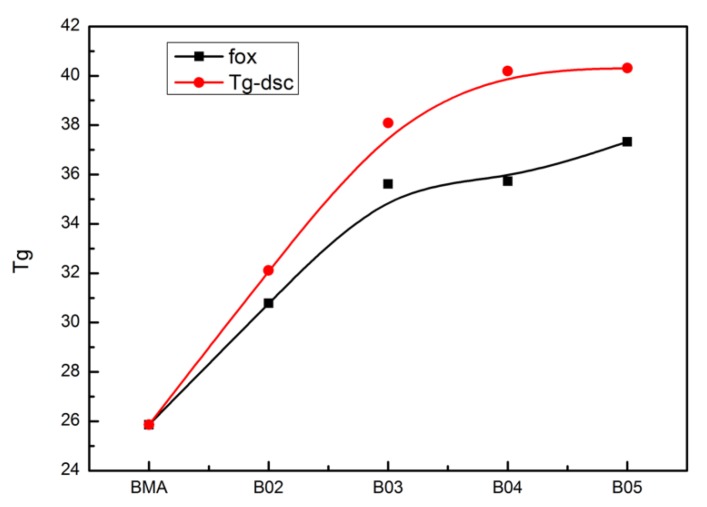
Tg of five hybrid systems with different molecular weights.

Glass transition is the cooperative motion of local segments, it is sensitive to the environment. Except for A01, the softening point of all phenolic resins is higher than T_g_ of PBMA in Figure 3a. With increasing molecular weight, it gradually moves to higher temperature. As for composites, all samples show only one glass transition in Figure 3b, so the composites might not have apparent heterogeneity. B01 has the lowest T_g_. With the increase of molecular weight, it increases rapidly at first and then rises slightly. In order to further understand this phenomenon. The T_g_ measured by DSC is compared with the T_g_ calculated by Fox equation. As shown in Figure 4, it is obvious that blends display higher T_g_ than that predicted by the Fox equation. This is mostly caused by the strong intermolecular interaction between fillers and matrix.

### 3.3. Molecular Dynamics of PBMA/PR Composites

Dynamic mechanical analysis (DMA) is widely used to detect dynamic mechanical properties and segmental dynamics of polymer composites. Tanδ is an important parameter to evaluate damping properties. Some studies on molecular dynamics have found that tanδ consists of three molecular motions (LSM, Sub-rouse, Rouse model) and the area of tanδ is influenced by them together [24,25,26]. Generally, the area where tanδ >0.3 is called the effective damping region [27,28]. In this paper, tanδ_max_ and tanδ peak area (TA) were used to evaluate the damping properties and other damping parameters of blends are listed in Table 2. It can be found in Table 2 that the addition of PR can effectively improve the damping properties of PBMA. Tanδ_max_ improves from 1.481 to 2.352, TA increases from 40.4 to 71.64. On the other hand, the molecular weight has a great influence on the damping property. Higher molecular weight leads to smaller improvement of damping properties. By adjusting the molecular weight properly, PR can improve the damping capacity of the polymer more effectively.

**Figure 5:**
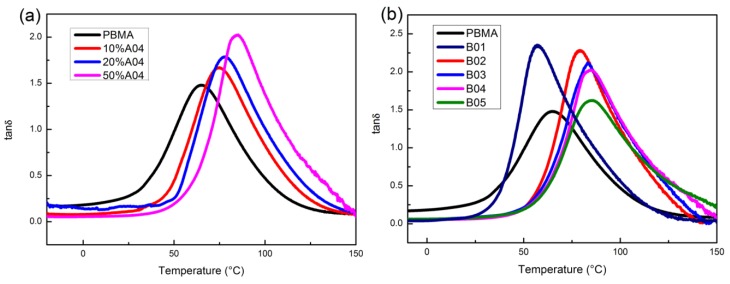
Temperature dependence of tanδ for (**a**) PBMA/A04 with different loadings, (**b**) PBMA, B01,B02, B03 B04, and B05.

The temperature dependence of tanδ of PBMA/A04 composites is shown in Figure 5a. With the increase of A04 content, tanδ peak position moves to higher temperature and tanδ_max_ increases. Hydrogen bonding plays a role in limiting molecular motion and increasing energy consumption at the same time. Figure 5b shows the variation of tanδ with PR molecular weight. A01 has a plasticizing effect on PBMA which is different from other PR, this can be attributed to the weaker interaction between A01 and PBMA. All composites show a higher tanδ_max_ than pure PBMA and the variation of tanδ peak position is consistent with those measured by DSC. It is worth noting that tanδ_max_ can be easily suppressed by a larger molecular weight. We think this is because that the PR with higher molecular weight has weaker motion ability that can reduce the energy consumption from hydrogen bonds.

**Figure 6:**
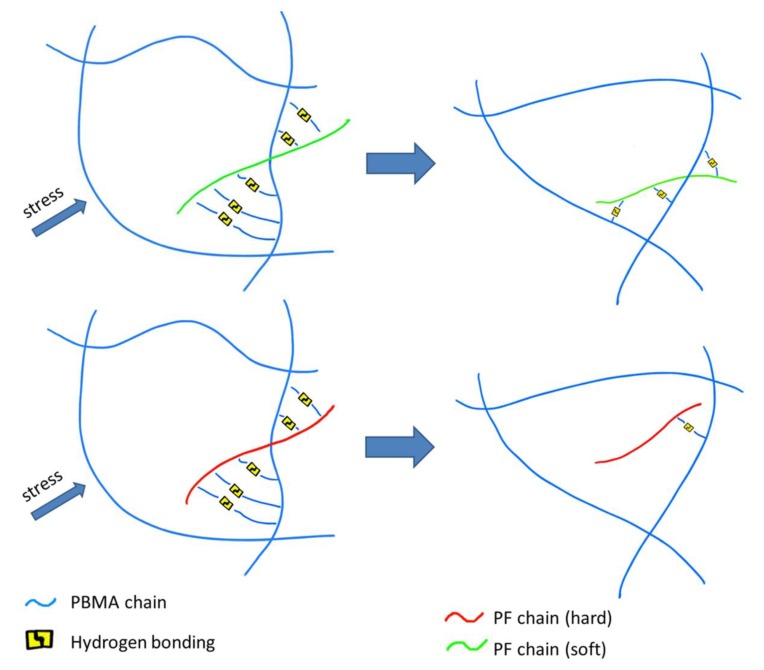
Schematic diagram of the recombination of hydrogen bonds as strain occurs.

In order to test our ideas, ∆T′ (Tg_-tanδ_-Tg_-dsc_) is used to evaluate the motion ability of PR in the glass transition region of PBMA/PR composites (T_g-dsc_ refers to the T_g_ of PR measured by DSC, T_g-tanδ_ refers to the T_g_ of PBMA/PR blends measured by DMA). As shown in Table 3, ∆T′ reduces with the increase of molecular weight. So the PR with higher molecular weight appears to be more rigid in the glass transition region. According to Radmard’s reports [29], flexible polymer chains are more conducive to the formation of hydrogen bonding. Therefore, lower molecular weight PR is more likely to form hydrogen bonds with PBMA and generate more energy loss through repeated formation and destruction of hydrogen bonding. As shown in Figure 6, a schematic diagram is used to represent this phenomenon. When the original hydrogen bonds are destroyed, the low molecular weight PR is more likely to form new ones with the matrix because of its flexibility.

**Figure 7:**
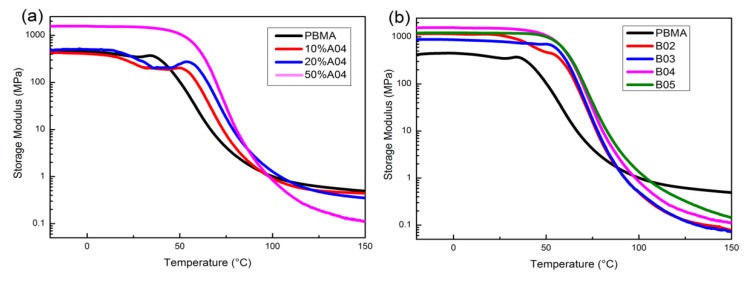
Temperature dependence of storage modulus for (**a**) PBMA/A04 with different loadings, (**b**) PBMA, B02, B03, B04, and B05.

Inspection of Figure 7a reveals the temperature dependence of the storage modulus (E′) for composites. In the glassy state, the blends have a higher modulus than pure PBMA. Hydrogen bonding plays a reinforcing role. As the temperature increases, the intermolecular force weakens and the fillers begin to soften. At this time, PR can reduce the entanglements in PBMA and the storage modulus of blends become lower than pure PBMA in the rubbery state.

On the other hand, the storage modulus of the composites improves with the increase of PR molecular weight in the rubbery state in Figure 7b. Storage modulus (G°_N_) of rubber platform is directly related to entanglements, as follow:

M_e_ = ρRT/G°_N_ (1)

where M_e_ represents entanglement molecular weight, ρ is the polymer density, R is the gas constant, and T is the temperature. That means the PBMA blended with larger molecular weight PR produce more entanglements and have less free volume. However, different scales of molecular motion have different responses to the space limited. The reduction in free volume can mainly confine the molecule motion with long scale and has little effect on small motion units such as segments corresponding to T_g_ [30,31]. This matches the phenomenon in Figure 5b. The weakening of large-scale molecule motion reduces tanδ_max_.

### 3.4. Intermolecular Interaction between PBMA and PR

**Figure 8:**
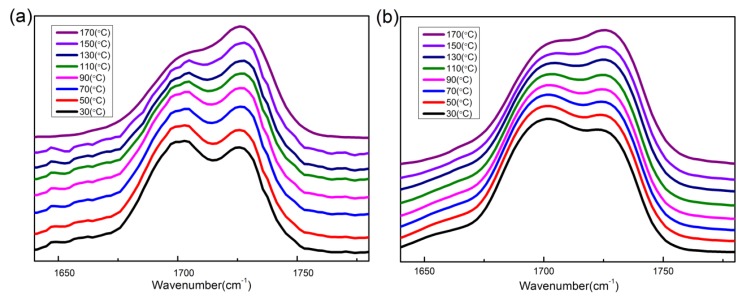
Temperature dependence of fourier transform infrared spectroscopy (FTIR) spectra in the carbonyl stretching region: (**a**) B04; (**b**) B01.

Figure 8 shows the infrared spectra of the carbonyl stretching region heating from 30 °C to 170 °C for B01 and B04. All spectra exhibit two bands at the carbonyl stretching region. One band centered at around 1728 cm^−1^ comes from the free carbonyl groups, another centered at around 1704 cm^−1^ corresponds to the hydrogen bonded carbonyl [32]. With increasing temperature, the intensity of hydrogen bonded carbonyl decreases, while the intensity of free carbonyl improves. To further study the temperature dependence of hydrogen bonds, the carbonyl stretching region was fitted with two Gaussian bands.

**Figure 9:**
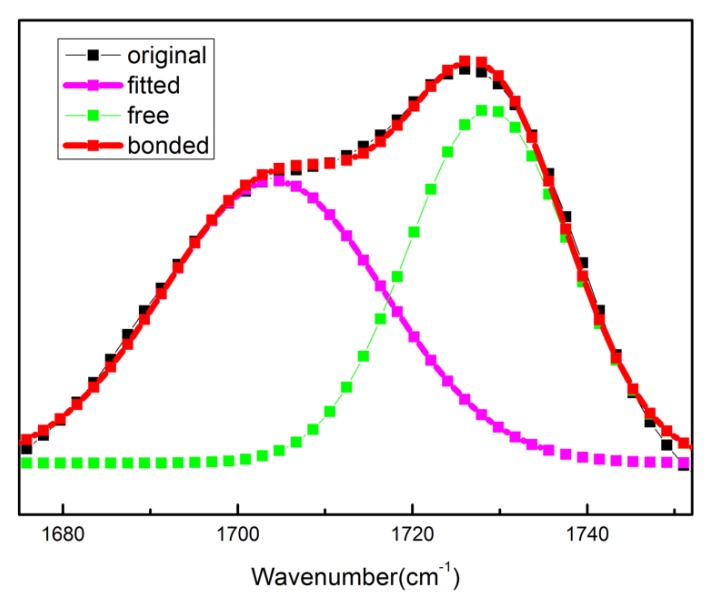
Curve fitting results in the carbonyl stretching region of B04 at 170 °C.

As shown in Figure 9, the carbonyl stretching region of B04 recorded at 170 °C was divided into two parts. The total area (A_T_) of the carbonyl region can be expressed by the following formula [33,34,35]:

A_T_ = A_B_ + A_F_ (2)

where A_B_ and A_F_ are the area of bonded and free carbonyl groups, respectively. The fraction of hydrogen-bonded carbonyl is given by:

X_B_ = A_B_/A_T_ (3)

**Figure 10:**
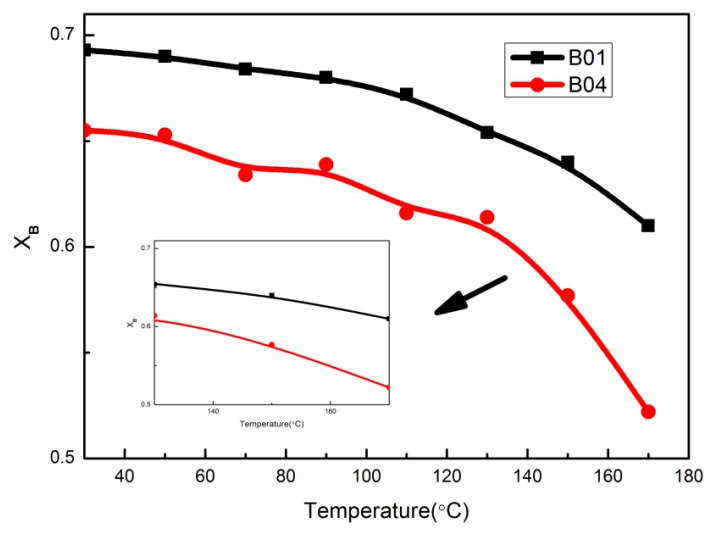
The fraction of hydrogen bonded carbonyl of the samples as a function of temperature.

Figure 10 shows the variation of X_B_ with temperature for B01 and B04. B01 forms more hydrogen bonds and has a weak dependence on temperature than B04, especially above 130 °C. This is consistent with our prediction. Combined with previous tests, we can draw a preliminary conclusion that lower molecular weight phenolic resin is more likely to form hydrogen bonds with PBMA because of its greater motion ability, which can effectively improve tanδ_max_.

## 4. Conclusions

The influence of PR molecular weight on the dynamic mechanical properties of PBMA/PR composites was investigated in this work. We used poly (butyl methacrylate) (PBMA) as matrix and a series of PR with different molecular weights as fillers for investigation. Solvent blending was used as a blending method. DSC and DMA found similar variation in T_g_. PR with higher molecular weight had weaker motion ability in the glass transition zone for PBMA/PR composites, which can weaken the energy dissipation from hydrogen bonding and reduce tanδ_max_. The variable temperature IR spectra found that PR with smaller molecular weight can form more hydrogen bonds with matrix and shows weaker temperature dependence. By analyzing the results, we found that increasing tanδ_max_ by hydrogen bonding energy requires a premise, the fillers need to have sufficient motion ability in the glass transition zone. The conclusion can be used as a guide for the preparation of high performance polymer damping materials.

## Figures and Tables

**Table 1:** Characteristics of the phenolic resins (PR) used.

Sample Code	Commercial Name	Mn	Mw	PDI	Softening Point (°C)
A01	558	538	751	1.40	24.14
A02	PF-2	680	1222	1.80	38
A03	8218	828	1607	1.94	57.19
A04	8219	930	1872	2.01	57.79
A05	M01	1272	4003	3.15	67.08

**Table 2:** The damping properties of PBMA/PR blends.

Sample Code	tanδ_max_	tanδ Peak Position (°C)	Temperate Range For tanδ > 0.3			TA (Tanδ > 1)
			T_1_ (°C)	T_2_ (°C)	∆T (°C)	
PBMA	1.481	65.08	27.45	109.13	81.8	40.4
B01	2.352	56.96	35.12	110.49	75.7	71.64
B02	2.282	79.14	53.6	126.77	73.7	68.52
B03	2.118	83.07	54.51	131.35	76.1	63.28
B04	2.023	84.38	55.93	138.62	82.9	60.49
B05	1.622	86.22	56.62	144.21	87.9	46.46

**Table 3:** The ∆T′ of B02, B03, B04, and B05.

Sample Code	Tg-tan δ (°C)	Sample Code	Tg-dsc (°C)	∆T′ (°C)
B02	79.14	A02	38	41.14
B03	83.07	A03	57.19	25.88
B04	84.38	A04	57.79	26.59
B05	86.22	A05	67.08	19.54
